# Kirrel3 homophilic adhesion function is required for male-directed aggression and vomeronasal acuity to urine chemosignals

**DOI:** 10.3389/fncel.2026.1926504

**Published:** 2026-09-08

**Authors:** Sydney Fearnley, Neelima Vaddadi, Emilie Dumontier, Jean-François Cloutier

**Affiliations:** 1 The Neuro, Montréal, QC, Canada; 2 Department of Anatomy and Cell Biology, McGill University, Montréal, QC, Canada; 3 Department of Neurology and Neurosurgery, McGill University, Montréal, QC, Canada

**Keywords:** accessory olfactory system, aggression, cell adhesion molecule, Kirrel, vomeronasal

## Abstract

The accessory olfactory system regulates social and reproductive behaviours in mice that are essential for survival. Proper function of this system depends on the accurate mapping of chemosensory information detected by vomeronasal sensory neurons (VSNs) into glomerular activation patterns. The formation of glomerular maps requires members of the Kirrel family of cell adhesion proteins, which mediate the coalescence of VSN axons into glomeruli. Disruption of Kirrel3 function, either through genetic deletion (*Kirrel3^−/−^*) or by impairing its homophilic interaction via a single amino acid substitution (*Kirrel3^Q128A^*), disrupts axonal glomerular map formation. Here, we compared the performance of the *Kirrel3^Q128A^* mice to *Kirrel3^−/−^* mice in behavioural assays involving social interactions. Similar to *Kirrel3^−/−^* mice, *Kirrel3^Q128A^* male mice exhibited reduced male–male aggression in the resident-intruder assay. Female *Kirrel3^Q128A^* mice lacked maternal aggression, although other maternal behaviors, including pup retrieval and nesting, remained intact. Urine preference assays revealed increased detection thresholds to both male and female urine in *Kirrel3^Q128A^* mice, indicating reduced vomeronasal sensitivity. However, response to non-social attractive and aversive odorants were unaffected. Notably, social novelty behavior was impaired in *Kirrel3^−/−^* mice but preserved in *Kirrel3^Q128A^* mice. Taken together, these results demonstrate that loss of Kirrel3-mediated adhesion is sufficient to recapitulate key deficits in vomeronasal function and VNO-dependent behaviors observed in Kirrel3 null mice, while selectively sparing certain aspects of social behavior.

## 1 Introduction

The accessory olfactory system plays a critical role in regulating a variety of social behaviors in mice, including aggression, mating, and maternal care (Bean and Wysocki, 1989; Haga-Yamanaka et al., 2014; Leypold et al., 2002; Stowers et al., 2002). These behaviors are driven by the detection of chemosignals from the environment by vomeronasal sensory neurons (VSNs) located in the vomeronasal organ (VNO), whose axons project to the accessory olfactory bulb (AOB) (Salazar et al., 2006). VSN axons expressing the same vomeronasal receptor coalesce to form approximately 6–30 homogeneously innervated glomeruli within the AOB (Belluscio et al., 1999; Del Punta et al., 2002; Martini et al., 2001; Rodriguez et al., 1999). This organization is spatially segregated with apical VSNs projecting axons to the anterior AOB, whereas basal VSNs project axons to the posterior AOB (Mori et al., 2000). Functionally, the posterior AOB has been implicated in regulating aggression toward pups and unfamiliar males, while the anterior AOB contributes to the regulation of mating and social hierarchy (Chamero et al., 2007, 2011; Haga-Yamanaka et al., 2014; Isogai et al., 2018; Pallé et al., 2020; Trouillet et al., 2019; Weiss et al., 2023).

The development of the accessory olfactory circuitry depends on coordinated mechanisms, including vomeronasal receptor expression, neuronal activity, and cell adhesion molecule function (Belluscio et al., 1999; Brignall et al., 2018; Hovis et al., 2012; Rodriguez et al., 1999). Among these, the Kirrel family of immunoglobulin-like cell adhesion proteins are differentially expressed on VSN axons and contribute to their precise targeting (Brignall et al., 2018). In particular, differential expression of Kirrel2 and Kirrel3 is thought to establish a molecular code that promotes the selective coalescence of like axons into glomeruli (Prince et al., 2013). Kirrel proteins mediate homophilic adhesion and do not heterodimerize due to incompatible binding interfaces (Wang et al., 2021). Mechanistically, Kirrel3 homodimerization is mediated by hydrophobic interactions within its first immunoglobulin domain, whereas Kirrel2 relies on hydrophilic interactions (Wang et al., 2021). A single amino acid substitution at position 128 (Q128A) disrupts Kirrel3 homodimerization, effectively abolishing its adhesive function.

Loss of Kirrel3-mediated adhesion results in disrupted glomerular organization in the AOB, characterized by fewer, enlarged glomeruli that are innervated by heterogeneous populations of VSN axons (Wang et al., 2021). These structural defects closely resemble those observed in Kirrel3 germline null mice, indicating that homophilic adhesion is essential for proper axonal coalescence and map formation (Prince et al., 2013). Consistent with this, Kirrel3 expression in basal VSNs is required for normal glomerular organization, vomeronasal sensitivity, and the expression of social behaviors such as aggression (Fearnley et al., 2026).

Despite these structural similarities, it remains unclear whether the specific disruption of Kirrel3 homophilic adhesion, independent of complete protein loss, is sufficient to alter behavior. In this study, we address this question using a knock-in mouse model carrying an amino acid substitution in Kirrel3 (Kirrel3Q128A), which selectively abolishes its homodimerization. We assess a range of behaviors associated with accessory and main olfactory system function in these mice and directly compare them to Kirrel3 null animals. This approach allows us to isolate the contribution of Kirrel3-mediated adhesion to the development and function of vomeronasal circuits and their associated behaviors.

## 2 Methods

### 2.1 Animals

*Kirrel3^−/−^* and *Kirrel3^Q128A^* mice have previously been described (Prince et al., 2013; Wang et al., 2021). Both mouse lines were maintained in a C57/Bl6 background. *Kirrel3^Q12A/Q128A^* mice are referred to as *Kirrel3^Q128A^* throughout the text. Castrated intruder C57/Bl6 males were purchased from Charles River. For all experimentations, littermate controls and mutants were compared blind. Control mice included wild-type and heterozygous animals.

### 2.2 Aggression behavior tests

A standard resident-intruder behavioral paradigm was used. Male test mice were single housed for at least 7 days prior to testing. During session 1, an age-matched castrated intruder mouse was introduced to the home cage of the test male for a 10-min period. Aggressive behaviors toward the intruder such as chasing, biting, and tail wagging were manually recorded. After 10 min, the intruder was removed. 24 h later, during session 2, the castrated intruder was reintroduced to the home cage of the test male. However, the intruder was swabbed with urine collected from intact breeder male mice. Urine was always collected fresh 1–2 h before testing and pooled from > 20 breeder males. Using a cotton swab, urine was applied to the intruder’s belly and back. The swabbed intruder was reintroduced to the test male’s home cage for a 10-min duration. Again, aggressive behaviors were manually recorded, and the intruder was removed after testing. All sessions were also video recorded.

A modified resident-intruder protocol was used to evaluate aggression in six-day postpartum female mice. A castrated male intruder was used for both sessions, but prior to addition of the intruder to the home cage, the pups were removed and placed into a separate transfer cage for the duration of testing. After each session, the intruder was removed and the pups returned to the home cage.

### 2.3 Urine dilution tests

Male and female urine was always collected fresh 1–2 h prior to testing. Urine was collected from >20 breeder mice and pooled to minimize individual variances. Collected urine (1:1) was diluted 10-fold in distilled water to produce 1:10, 1:100, 1:1000, and 1:10000 dilutions. Testing occurred in a (30 × 18 × 13 cm) transfer cage with no bedding and cleaned with 70% ethanol between each testing session. For each test, a cotton swab containing urine was taped to one side of the transfer cage with a control water cotton swab taped to the other side. Testing started with the most diluted urine swab (1:10000) up to undiluted urine (1:1) in 2-min intervals. Time spent sniffing each swab was manually recorded, as well as video recorded with an overhead camera. Preference index was calculated as % time spent sniffing the urine swab minus time spent sniffing the water swab, divided by total sniffing time.

### 2.4 Peanut butter dilution test

To make a peanut butter stock solution (1:1), peanut butter was dissolved in peanut oil at a 30% concentration. The stock solution was then diluted 10-fold in peanut oil to make 1:10, 1:100, 1:1000, and 1:10000 dilutions. Testing occurred in a (30 × 18 × 13 cm) transfer cage with no bedding and cleaned with 70% ethanol between animals. Testing started with the most diluted peanut butter swab (1:10000) up to least dilute (1:1) in 2-min intervals. Sniffing time of both cotton swabs was measured manually, and all testing was filmed using an overhead camera. Preference index was calculated as % time spent sniffing the peanut butter swab minus time spent sniffing the peanut oil swab, divided by total sniffing time.

### 2.5 Repellent test

Pure 2-methylbutyric acid was diluted in distilled water to make 1:10, 1:100, 1:1000, and 1:10000 dilutions. Testing occurred in a (30 × 18 × 13 cm) transfer cage with no bedding and cleaned with 70% ethanol. The testing transfer cage was measured with a ruler and divided into thirds and marked underneath with tape. Testing started with the most diluted swab (1:10000) up to least dilute (1:1) in 2-min intervals. Because 2-methylbutyric acid is a highly volatile and aversive odorant, time spent in the third of the cage closest to the acid swab was quantified instead of direct swab sniffing time. Preference index was calculated as % time spent in the third of the cage closest to the acid swab minus time spent in the remaining two thirds of the testing cage, divided by total sniffing time.

### 2.6 Pup retrieval behavior test

Testing occurred in the dam’s home cage on postnatal days 0–3 after birth (P0-P3). Briefly, the dam was removed and temporarily placed in a separate transfer cage. Three pups were placed in the corners of the home cage opposite the nest. The dam was then reintroduced to the cage and the latency to retrieve the pups back to the test was measured. All testing was also recorded with an overhead camera.

### 2.7 Nesting behavior test

A standard nesting protocol was followed (Deacon, 2006). Testing occurred in the dam’s home cage on postnatal days 0–3 after birth (P0-P3). 1–3 days before birth, all previous nesting material was removed and replaced with two fresh pieces of nestlet. Each nestlet weighed 2–3 grams. On testing days, a picture of the nest was taken and scored on a scale of 1–5, with 5 being the highest score. A score of 5 meant all nestlet was shredded, all nestlet pieces were contained to the nest, all pups were inside the nest and covered by nestlet. For each condition not met, a point was removed.

### 2.8 Quantification and statistical analysis

All behavior tests were manually quantified using BORIS software (Friard and Gamba, 2016). Statistical analyses were performed with GraphPad Prism 8. Data is represented as mean ± SEM. Statistical tests and *n* for each analysis are stated in the figure captions. Detailed statistical tests performed and their results can be found in Supplementary Table S1.

## 3 Results

### 3.1 Kirrel3 homophilic adhesion function is required for male–male aggression

Aggressive behavior in mice is a social behavior that is strongly regulated by the accessory olfactory system, which detects social chemosignals present in urine (Bean and Wysocki, 1989; Stowers et al., 2002; Trouillet et al., 2019). In particular, aggression-triggering cues, such as major urinary proteins, are detected by VSNs in the basal region of the VNO (Chamero et al., 2007; Maruniak et al., 1989). Previous studies have shown that germline deletion of Kirrel3, or its specific ablation in basal VSNs, alters glomeruli formation and reduces male–male aggression (Prince et al., 2013; Fearnley et al., 2026). However, it remains unclear whether selective disruption of Kirrel3 homophilic adhesion is sufficient to produce similar behavioral effects.

**Figure 1:**
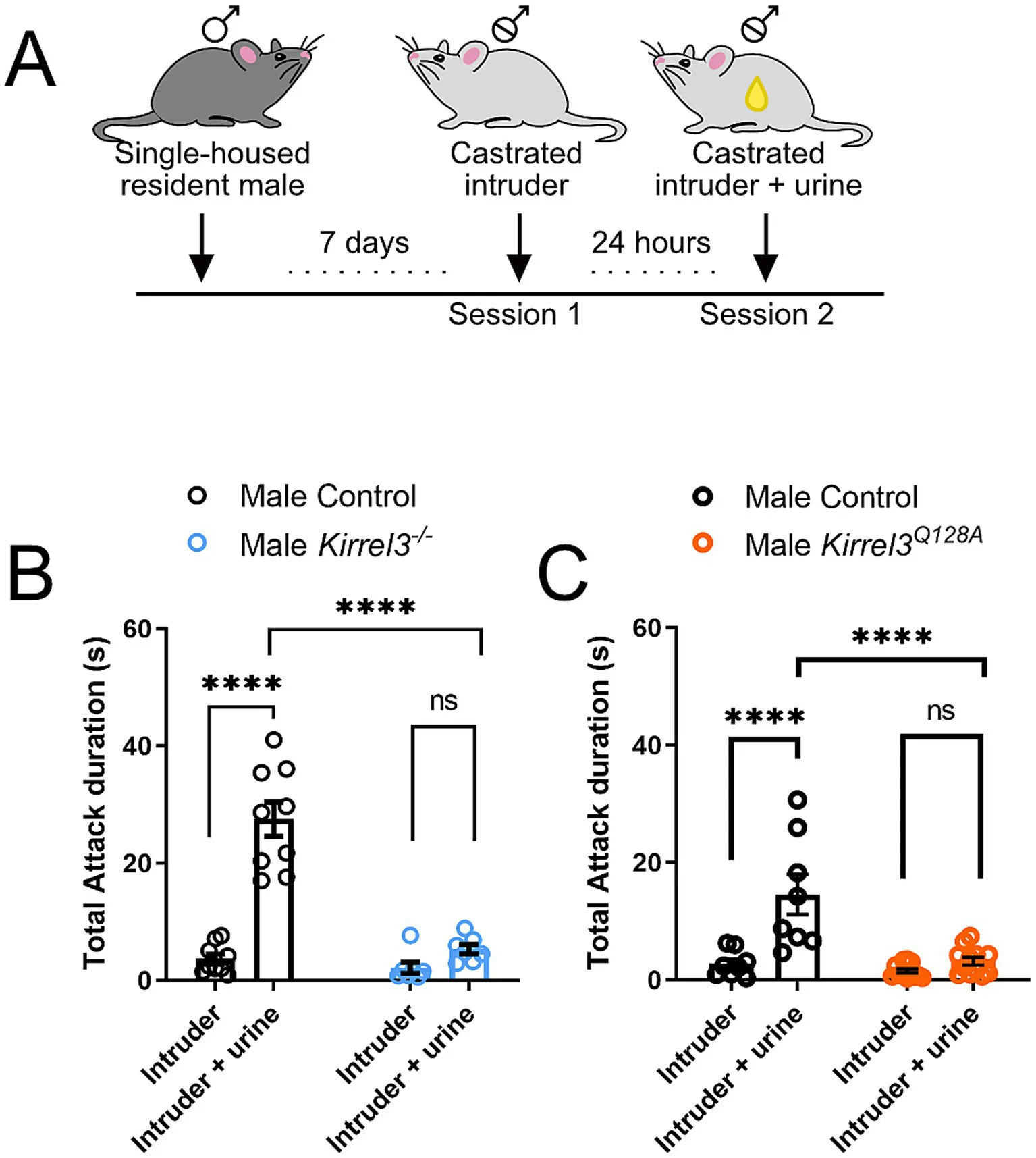
Kirrel3 homophilic adhesion function is required for male–male aggression. **(A)** Schematic of the resident-intruder test. **(B)** Quantification of attack duration for the resident-intruder test in control and *Kirrel3^−/−^* male mice. While attack duration during session 1 was low for both genotypes (control: 3.66 ± 0.82 s, *Kirrel3^−/−^*: 2.12 ± 0.04 s), only control animals showed significantly higher attack duration during session 2 (control: 27.52 ± 2.92 s, *Kirrel3^−/−^*: 5.30 ± 0.79 s). Control sessions: ****p < 0.0001, Mutant sessions: ns *p* = 0.3380; Genotypes: ****p < 0.0001; 2-way ANOVA with Bonferroni correction; *F* (1, 14) = 31.51. **(C)** Quantification of attack duration for the resident-intruder test in control and *Kirrel^Q128A^* male mice. Attack duration during session 1 was low for both genotypes (control: 2.78 ± 0.80 s, *Kirrel^Q128A^*: 1.54 ± 0.34 s), and only control animals showed significantly higher attack duration during session 2 (control: 14.57 ± 3.40 s, *Kirrel^Q128A^*: 3.18 ± 0.65 s). Control sessions: ****p < 0.0001, Mutant sessions: ns *p* = 0.5965; Genotypes: ****p < 0.0001; 2-way ANOVA with Bonferroni correction; *F* (1, 18) = 13.55. Data is represented as mean ± SEM. *n* = 9 control and 7 *Kirrel3^−/−^* mice for **(B)** and *n* = 8 control and 7 *Kirrel3^Q128A^* mice for **(C)**.

To address this, we assessed aggressive behavior in male *Kirrel3^−/−^* and *Kirrel3^Q128A^* mice using the resident-intruder test (Figure 1A), a well-established paradigm to measure urine-evoked aggression (Bean and Wysocki, 1989; Chamero et al., 2011; Connor, 1974; Leypold et al., 2002; Maruniak et al., 1989; Stowers et al., 2002). For session 1, a castrated intruder was introduced into the home cage of the resident male. Under these conditions, both control and *Kirrel3^−/−^* mice displayed minimal aggression (control: 3.66 ± 0.82 s, *Kirrel3^−/−^*: 2.12 ± 0.04 s, Figure 1B). When the same intruder was swabbed with urine collected from intact breeder males, control mice displayed a marked increase in aggressive behaviors, including chasing, tail wagging, and biting (27.52 ± 2.92 s, *p* < 0.0001, Figure 1B). In contrast, *Kirrel3^−/−^* males did not exhibit increased aggression towards the urine-swabbed intruder (5.30 ± 0.79 s, *p* = 0.3380, Figure 1B). This result replicates previous reports demonstrating reduced male–male aggression in *Kirrel3^−/−^* mice (Hisaoka et al., 2018; Prince et al., 2013).

We next examined *Kirrel3^Q128A^* males to determine whether disrupting Kirrel3 homophilic adhesion is sufficient to impair aggression. Similar to *Kirrel3^−/−^* mice, *Kirrel3^Q128A^* males did not show increased attack behavior in response to urine-swabbed intruders (session 1 *Kirrel3^Q128A^*: 1.54 ± 0.34 s, session 2 *Kirrel3^Q128A^*: 3.18 ± 0.65 s, *p* = 0.5965, Figure 1C) whereas control mice exhibited a significant increase in aggressive behaviors (session 1 control: 2.78 ± 0.80 s, session 2 control: 14.57 ± 3.40 s, *p* < 0.0001, Figure 1C). These results indicate that Kirrel3 homophilic adhesion is necessary for the normal expression of urine-evoked male aggression in the resident-intruder test.

### 3.2 Maternal aggression is decreased, but other maternal behaviors remain unchanged in Kirrel3^Q128A^ mice

**Figure 2:**
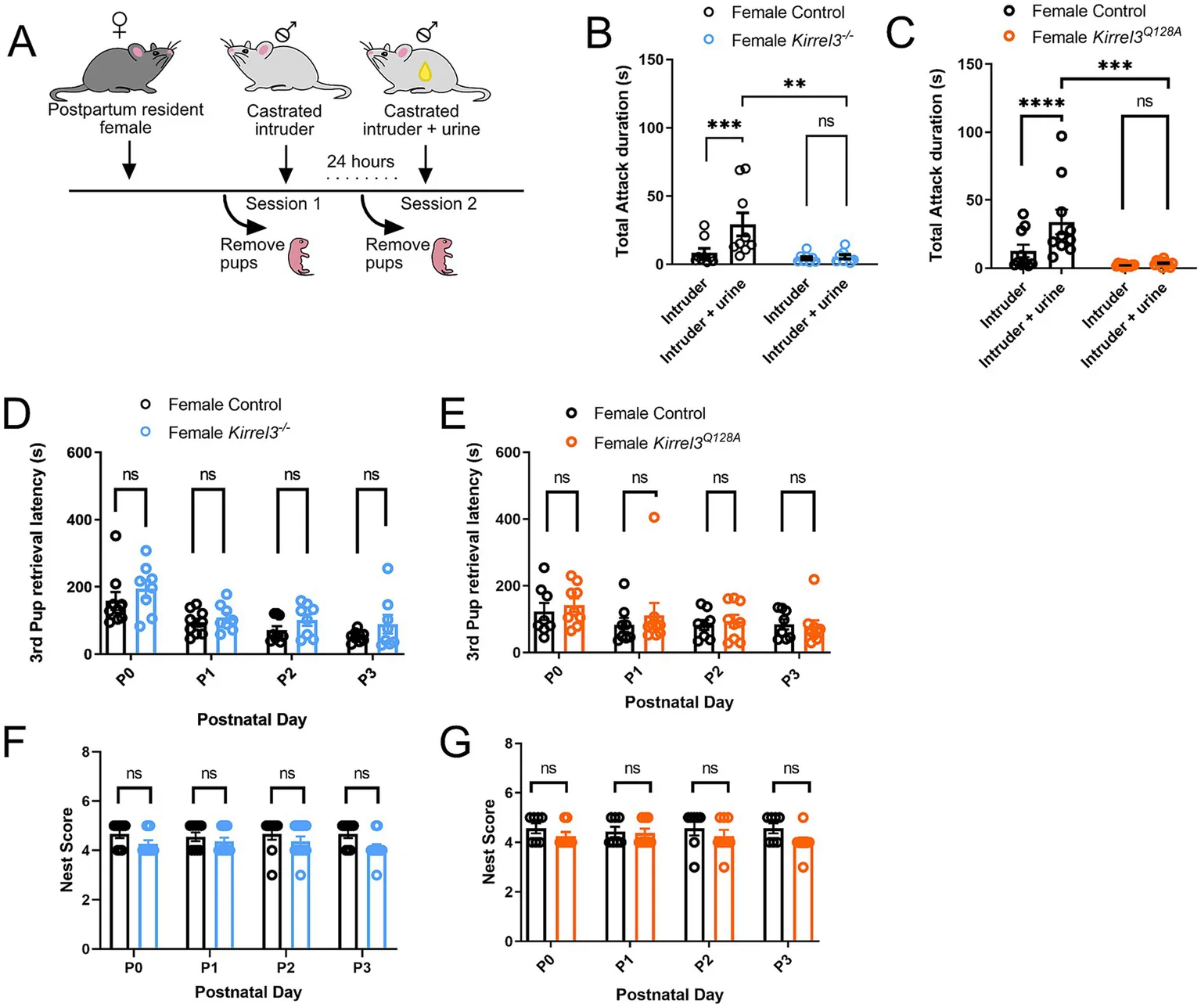
Kirrel3 homophilic adhesion function is required for maternal aggression. **(A)** Schematic for the maternal aggression test. **(B)** Attack duration for the maternal aggression test in control and *Kirrel3^−/−^* postpartum female mice. Attack duration during session 1 was low for both genotypes (control: 8.44 ± 3.18 s, *Kirrel3^−/−^*: 4.48 ± 1.18 s), and only control females showed significantly higher attack duration during session 2 (control: 29.18 ± 8.45 s, *Kirrel3^−/−^*: 5.52 ± 1.56 s). Control sessions: ***p = 0.0004, Mutant sessions: ns *p* > 0.9999; Genotypes: **p = 0.0037; 2-way ANOVA with Bonferroni correction; *F* (1, 15) = 4.995. **(C)** Quantification of attack duration for the maternal aggression test in control and *Kirrel3^Q128A^* postpartum female mice. Attack duration during session 1 was low for both genotypes (control: 12.57 ± 4.55 s, *Kirrel3^Q128A^*: 2.10 ± 0.25 s), and only control females showed significantly higher attack duration during session 2 (control: 33.90 ± 8.99 s, *Kirrel3^Q128A^*: 3.54 ± 0.63 s). Control sessions: ****p < 0.0001, Mutant sessions: ns p > 0.9999; Genotypes: ***p = 0.0001; 2-way ANOVA with Bonferroni correction; *F* (1, 19) = 10.90. **(D)** Latency to retrieve the third pup back to the nest on postnatal days 0–3 (P0-P3) in control and *Kirrel3^−/−^* postpartum female mice. No difference was observed between genotypes on each postnatal day (P0 control: 158.44 ± 26.70 s, P0 *Kirrel3^−/−^*: 194.00 ± 26.47 s; P1 control: 94.33 ± 12.27 s, P1 *Kirrel3^−/−^*: 108.37 ± 14.08 s; P2 control: 71.88 ± 12.10 s, P2 *Kirrel3^−/−^*: 101.87 ± 16.99 s; P3 control: 52.55 ± 5.13 s, P3 *Kirrel3^−/−^*: 89.50 ± 27.90 s). For P0 and P1: ns p > 0.9999; P2 ns *p* = 0.6971; P3 ns *p* = 9,262. 2-way ANOVA with Bonferroni correction; F (1, 15) = 3.635. **(E)** Pup retrieval latency to retrieve the third pup back to the nest on postnatal days 0–3 (P0-P3) in control and *Kirrel3^Q128A^* postpartum female mice. No difference was observed between genotypes on each postnatal day (P0 control: 122.87 ± 25.61 s, P0 *Kirrel3^Q128A^*: 142.00 ± 20.02 s; P1 control: 83.00 ± 20.22 s, P1 *Kirrel3^Q128A^*: 110.77 ± 37.24 s; P2 control: 82.87 ± 15.04 s, P2 *Kirrel3^Q128A^*: 94.44 ± 18.44 s; P3 control: 84.50 ± 15.17 s, P3 *Kirrel3^Q128A^*: 77.77 ± 18.60 s). For P0, P1, P2, and P3: ns *p* > 0.9999. 2-way ANOVA with Bonferroni correction; F (1, 15) = 0.5016. **(F)** Average nest score during the nest building behavior test in control and *Kirrel3^−/−^* postpartum female mice. No difference was observed between genotypes on each postnatal day (P0 control: 4.66 ± 0.16, P0 *Kirrel3^−/−^*: 4.27 ± 0.14; P1 control: 4.55 ± 0.17, P1 *Kirrel3^−/−^*: 4.36 ± 0.15; P2 control: 4.66 ± 0.23, P2 *Kirrel3^−/−^*: 4.36 ± 0.20; P3 control: 4.66 ± 0.16, P3 *Kirrel3^−/−^*: 4.09 ± 0.16). P0: ns *p* = 0.3939; P1 ns *p* = 0.1919; P2 ns *p* = 0.3030, P3: ns *p* = 0.5758. 2-way ANOVA with Bonferroni correction; *F* (1, 18) = 4.055. **(G)** Average nest score during the nest building behavior test in control and *Kirrel3^Q128A^* postpartum female mice. No difference was observed between genotypes on each postnatal day (P0 control: 4.57 ± 0.20, P0 *Kirrel3^Q128A^*: 4.25 ± 0.16; P1 control: 4.42 ± 0.20, P1 *Kirrel3^Q128A^*: 4.37 ± 0.18; P2 control: 4.71 ± 0.29, P2 *Kirrel3^Q128A^*: 4.25 ± 0.25; P3 control: 4.57 ± 0.20, P3 *Kirrel3^Q128A^*: 4.00 ± 0.18). P0: ns *p* = 0.3214; P1 ns *p* = 0.0535; P2 ns p = 0.3214, P3: ns *p* = 0.5714. 2-way ANOVA with Bonferroni correction; *F* (1, 13) = 2.183. Data is represented as mean ± SEM. *n* = 9 control and 8 *Kirrel3^−/−^* mice for **(B,D,F)**, *n* = 10 control and 11 *Kirrel3^Q128A^* mice for **(C)**, and *n* = 8 control and 9 *Kirrel3^Q128A^* mice for **(E,G)**.

Territorial aggression towards male conspecifics is not limited to males. Postpartum female mice also display robust aggression toward unfamiliar males to protect their offspring from infanticide (Gandelman, 1972; Wolff, 1985). To assess whether the aggression deficit observed in *Kirrel3* mutant males extends to females, we performed a modified resident-intruder assay using postpartum females (Figure 2A).

During session 1, introduction of a castrated male intruder into the female’s home cage elicited minimal aggression from both control and *Kirrel3^−/−^* females (control: 8.44 ± 3.18 s, *Kirrel3^−/−^*: 4.48 ± 1.18 s, Figure 2B). In session 2, the same intruder was swabbed with urine from sexually experienced males and reintroduced. Under these conditions, control females showed a significant increase in attack duration compared to session 1 (control: 29.18 ± 8.45 s, *p* = 0.0004, Figure 2B). However, *Kirrel3^−/−^* females showed little to no increase in aggression toward the urine-swabbed intruder (*Kirrel3^−/−^*: 5.52 ± 1.56 s, *p* > 0.9999, Figure 2B). Similarly, *Kirrel3^Q128A^* mutant females showed no significant increase in aggression towards the swabbed intruder (control session 1: 12.579 ± 4.558 s, control session 2: 33.90 ± 8.99 s, *p* < 0.0001; *Kirrel3^Q128A^* session 1: 2.100 ± 0.252 s, *Kirrel3^Q128A^* session 2: 3.54 ± 0.63 s, *p* > 0.9999; Figure 2C). These results demonstrate that both Kirrel3 expression and its homophilic adhesion function are essential for normal maternal aggression.

In addition to being expressed in VSNs, Kirrel3 is expressed in olfactory sensory neurons and influences glomerular organization in the main olfactory bulb (Serizawa et al., 2006). Disruption of signaling in olfactory sensory neurons impairs multiple aspects of maternal behavior, including pup retrieval, nest building, and aggression (Dudas et al., 2024; Mandiyan et al., 2005; Wang and Storm, 2011). We therefore examined whether pup retrieval and nest building behaviors are also affected in *Kirrel3* mutant mice.

Pup retrieval was quantified as the latency to return three pups to the nest during post-natal days 0–3 (P0 to P3; Figures 2D,E). Nest building was evaluated over the first four postnatal days using a standardized 1–5 scoring scale (Figures 2F,G). Neither *Kirrel3^−/−^* nor *Kirrel3^Q128A^* female mice differed from controls in pup retrieval latency (control and *Kirrel3^−/−^*: P0 and P1 *p* > 0.9999, P2 *p* = 0.6971, P3 *p* = 9,262, control and *Kirrel3^Q128A^*: P0, P1, P2, P3 *p* > 0.9999, Figures 2D,E) or nest quality (control and *Kirrel3^−/−^*: P0 *p* = 0.3939, P1 *p* = 0.1919, P2 *p* = 0.3030, P3 *p* = 0.5758; control and *Kirrel3^Q128A^*: P0 *p* = 0.3214, P1 *p* = 0.0535, P2 *p* = 0.3214, P3 *p* = 0.5714, Figures 2F,G). Together, these results demonstrate that disruption of Kirrel3 function selectively impairs maternal aggression without affecting other core maternal behaviors, suggesting that the reduced aggression likely stems from malfunction of the accessory olfactory system.

### 3.3 *Kirrel3^Q128A^* mice display decreased vomeronasal acuity to urine chemosignals

**Figure 3:**
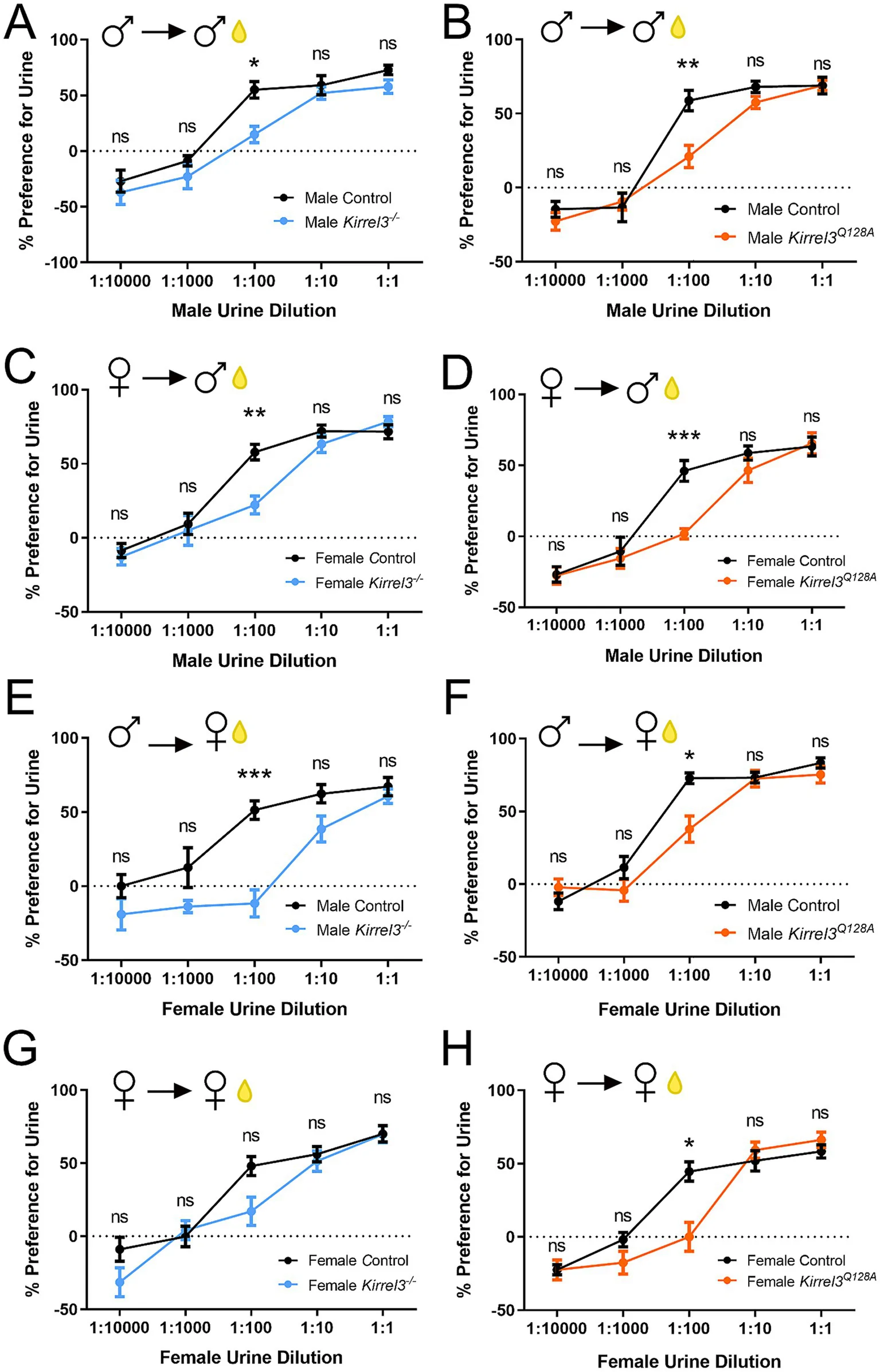
*Kirrel3^−/−^* and *Kirrel3^Q128A^* mice display decreased vomeronasal acuity to urine chemosignals. **(A)** Urine dilution test for male control and *Kirrel3^−/−^* mice sniffing male urine. Percentage preference was calculated as [(time spent sniffing urine swab – time spent sniffing water swab) / total sniffing time]. At the 1:100 urine dilution, *Kirrel3^−/−^* mice showed a significantly lower preference for the urine swab over the water swab compared to control littermates (control: 55.05 ± 7.40 percent, *Kirrel3^−/−^*: 14.95 ± 7.35 percent). 1:100 dilution: *p = 0.0123; all other dilutions: ns *p* > 0.9999; 2-way ANOVA with Bonferroni correction; *F* (1, 12) = 6.973. **(B)** Urine dilution test for male control and male *Kirrel3^Q128A^* mice sniffing male urine. While control mice displayed a positive preference for the urine cotton swab at the 1:100 dilution, *Kirrel3^Q128A^* mice did not prefer the urine swab until the 1:10 dilution (control: 55.05 ± 7.40 percent, *Kirrel3^Q128A^*: 14.95 ± 7.35 percent). 1:100 dilution: **p = 0.0079; All other dilutions: ns *p* > 0.9999; 2-way ANOVA with Bonferroni correction; *F* (1, 19) = 6.897. **(C)** Urine dilution test for female control and female *Kirrel3^−/−^* mice sniffing male urine. At the 1:100 urine dilution, *Kirrel3^−/−^* mice showed a significantly lower preference for the urine swab over the water swab compared to control littermates (control: 57.86 ± 5.23 percent, *Kirrel3^−/−^*: 22.134 ± 6.00 percent). 1:100 dilution: **p = 0.0018; All other dilutions: ns *p* > 0.9999; 2-way ANOVA with Bonferroni correction; *F* (1, 17) = 4.411. **(D)** Urine dilution test for female control and female *Kirrel3^Q128A^* mice sniffing male urine. While control mice displayed a positive preference for the urine cotton swab at the 1:100 dilution, *Kirrel3^Q128A^* mice did not prefer the urine swab until the 1:10 dilution (control: 46.10 ± 7.33 percent, *Kirrel3^Q128A^*: 1.82 ± 3.58 percent). 1:100 dilution: ***p = 0.0009; All other dilutions: ns *p* > 0.9999; 2-way ANOVA with Bonferroni correction; *F* (1, 15) = 4.054. **(E)** Urine dilution test for male control and male *Kirrel3^−/−^* mice sniffing female urine. At the 1:100 urine dilution, *Kirrel3^−/−^* mice showed a significantly lower preference for the urine swab over the water swab compared to control littermates (control: 51.28 ± 6.26 percent, *Kirrel3^−/−^*: −11.73 ± 9.13 percent). 1:100 dilution: ***p = 0.0002; All other dilutions: ns *p* > 0.9999; 2-way ANOVA with Bonferroni correction; F (1, 18) = 32.30. **(F)** Urine dilution test for male control and male *Kirrel3^Q128A^* mice sniffing female urine. While control mice displayed a positive preference for the urine cotton swab at the 1:100 dilution, *Kirrel3^Q128A^* mice did not prefer the urine swab until the 1:10 dilution (control: 72.76 ± 2.67 percent, *Kirrel3^Q128A^*: 37.80 ± 9.05 percent). 1:100 dilution: *p = 0.0145; All other dilutions: ns *p* > 0.9999; 2-way ANOVA with Bonferroni correction; *F* (1, 20) = 5.466. **(G)** Urine dilution test for female control and *Kirrel3^−/−^* mice sniffing female urine. At the 1:100 urine dilution, *Kirrel3^−/−^* mice appeared to have a lower preference for the urine swab over the water swab compared to control littermates, but this change did not reach statistical significance; (control: 48.08 ± 6.48 percent, *Kirrel3^−/−^*: 17.12 ± 9.64 percent). 1:100 dilution: ns *p* = 0.1002; All other dilutions: ns *p* > 0.9999; 2-way ANOVA with Bonferroni correction; F (1, 15) = 2.833. **(H)** Urine dilution test for female control and *Kirrel3^Q128A^* mice sniffing female urine. While control mice displayed a positive preference for the urine cotton swab at the 1:100 dilution, *Kirrel3^Q128A^* mice did not prefer the urine swab until the 1:10 dilution (control: 44.61 ± 6.59 percent, *Kirrel3^Q128A^*: 0.10 ± 9.86 percent). 1:100 dilution: *p = 0.0130; All other dilutions: ns *p* > 0.9999; 2-way ANOVA with Bonferroni correction; *F* (1, 15) = 2.773. Data is represented as mean ± SEM. *n* = 8 male control and 6 male *Kirrel3^−/−^* mice for **(A)**, *n* = 9 male control and 12 male *Kirrel3^Q128A^* mice for **(B,F)**, *n* = 10 male control and 10 male *Kirrel3^−/−^* mice for **(E)**, *n* = 9 female control and 8 female *Kirrel3^−/−^* mice for **(C,G)**, *n* = 9 female control and 9 female *Kirrel3^Q128A^* mice for **(D,H)**.

The reduced male-directed aggression observed in *Kirrel3^−/−^* and *Kirrel3^Q128A^* mice could reflect impaired vomeronasal system function. Consistent with this possibility, selective loss of Kirrel3 in basal VSNs has been associated with decreased sensitivity to urinary chemosignals (Fearnley et al., 2026). To assess vomeronasal acuity, we measured investigation preferences for swabs containing increasing dilutions of urine versus water. Control male mice exhibited a clear preference for male urine over water at a dilution of 1:100 (*Kirrel3* control: 55.05 ± 7.40 percent, *Kirrel3^Q128A^* control: 58.82 ± 6.92 percent, Figures 3A,B). In contrast, *Kirrel3^−/−^* and *Kirrel3^Q128A^* males showed significantly reduced preference at this urine dilution compared to control littermates, indicating decreased sensitivity to a low concentration of urine chemosignals (*Kirrel3^−/−^*: 14.95 ± 7.35 percent, *p* = 0.0123; *Kirrel3^Q128A^*: 21.05 ± 7.54 percent, *p* = 0.0079, Figures 3A,B). At higher concentrations (1:10 dilution or undiluted urine), mutant males displayed preferences comparable to controls (*Kirrel3^−/−^ p* > 0.9999, *Kirrel3^Q128A^ p* > 0.9999), suggesting that the deficit is limited to detection of chemosignals at a lower concentration.

To determine whether this phenotype was sex-specific, we performed the same assay in female mice. Both *Kirrel3^−/−^* (control: 57.86 ± 5.23 percent, *Kirrel3^−/−^*: 22.134 ± 6.00 percent, *p* = 0.0018, Figure 3C) and *Kirrel3^Q128A^* (control: 46.10 ± 7.33 percent, *Kirrel3^Q128A^*: 1.82 ± 3.58 percent, *p* = 0.0009, Figure 3D) female mice showed a similar reduction in preference at lower urine concentrations, indicating that the vomeronasal acuity deficit is not restricted to males.

Because male and female urine contain distinct classes of chemosignals, including major urinary proteins and sulfated steroids, and activate partially non-overlapping populations of glomeruli in in the AOB, we next asked whether the observed vomeronasal acuity deficit was stimulus-specific (Hammen et al., 2014; Kaur et al., 2014; Meeks et al., 2010; Nodari et al., 2008). We therefore repeated the dilution assay using female urine. As with male urine, *Kirrel3* mutant mice required higher concentrations of female urine to exhibit a preference over water. Male *Kirrel3^−/−^* (control: 51.28 ± 6.26 percent, *Kirrel3^−/−^*: −11.73 ± 9.13 percent, *p* = 0.0002, 1:100 urine dilution, Figure 3E) and *Kirrel3^Q128A^* (control: 72.76 ± 2.67 percent, *Kirrel3^Q128A^*: 37.80 ± 9.05 percent, *p* = 0.0145, 1:100 urine dilution, Figure 3F) did not show significant preference until up to a ~ 10-fold higher concentration relative to controls. *Kirrel3^Q128A^* females also displayed reduced sensitivity to female urine at a lower concentration of urine (control: 44.61 ± 6.59 percent, *Kirrel3^Q128A^*: 0.10 ± 9.86 percent, *p* = 0.0130, 1:100 urine dilution, Figure 3H). *Kirrel3^−/−^* females showed a small decrease in preference for female urine compared to controls, which did not reach statistical significance (control: 48.08 ± 6.48 percent, *Kirrel3^−/−^*: 17.12 ± 9.64 percent, *p* = 0.1002, 1:100 urine dilution, Figure 3G). Taken together, these results demonstrate that disruption of Kirrel3-mediated homophilic adhesion impairs vomeronasal acuity and is sufficient to affect vomeronasal acuity independently of the sex of the mouse, or of the type of urine detected.

### 3.4 Acuity to main olfactory odorants is unaffected in *Kirrel3^Q128A^* mice

The main olfactory system detects a broad range of odorants, including cues involved in food finding and predator avoidance (Horio et al., 2019; Nguyen et al., 2023). Since Kirrel3 is expressed in olfactory sensory neurons and the Q128A substitution disrupts homophilic adhesion in all Kirrel3-expressing cell types, we asked whether Kirrel3 function is required for odor detection mediated by the main olfactory system.

**Figure 4:**
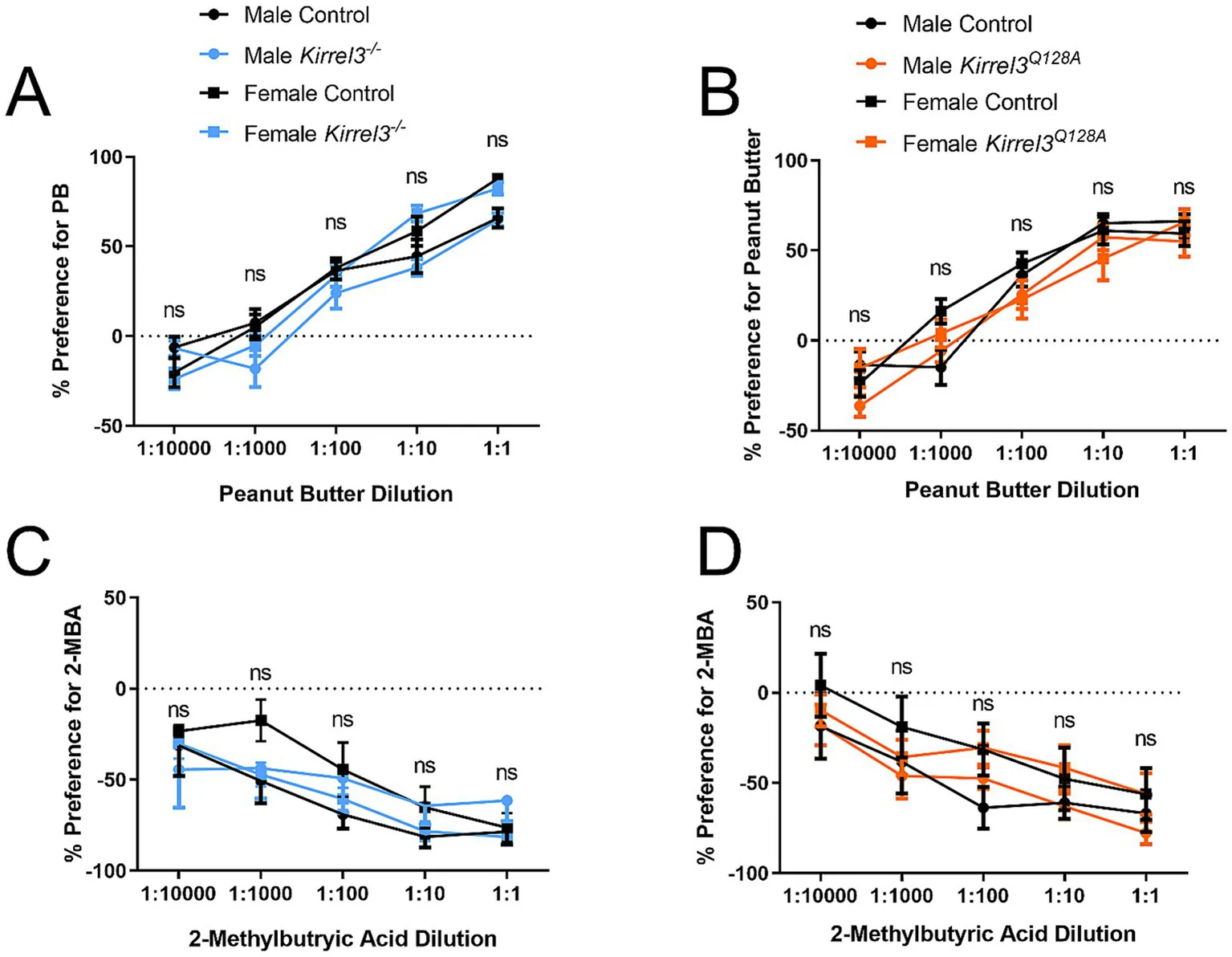
Acuity to odorants detected by the main olfactory system remains unchanged in *Kirrel3^−/−^* and *Kirrel3^Q128A^* mice. **(A)** Peanut butter dilution test for male and female control and *Kirrel3^−/−^* mice. No difference between sexes or genotypes was observed. All dilutions: ns p > 0.9999; 2-way Anova with Bonferroni correction; *F* (3, 26) = 2.929. **(B)** Peanut butter dilution test for control and *Kirrel3^Q128A^* mice. No difference between sexes or genotypes was observed. All dilutions: ns p > 0.9999; 2-way Anova with Bonferroni correction; *F* (3, 33) = 1.625. **(C)** 2-Methylbutyric acid dilution test for control and *Kirrel3^−/−^* mice. No difference between sexes or genotypes was observed. All dilutions: Ns *p* > 0.9999; 2-way ANOVA with Bonferroni correction; *F* (3, 23) = 0.8148. **(D)** 2-Methylbutyric acid dilution test for control and *Kirrel3^Q128A^* mice. No difference between sexes or genotypes was observed. All dilutions: ns *p* > 0.9999; 2-way ANOVA with Bonferroni correction; *F* (3, 28) = 1.319. Data is represented as mean ± SEM. *n* = 8 male control, 6 male *Kirrel3^−/−^, n* = 9 female control and 7 female *Kirrel3^−/−^* mice for **(A)**, *n* = 8 male control, 7 male *Kirrel3^−/−^, n* = 5 female control and 7 female *Kirrel3^−/−^* mice for **(B)**, *n* = 9 male control, 11 male *Kirrel3^Q128A^, n* = 9 female control and 8 female *Kirrel3^Q128A^* mice for **(C)**, and *n* = 6 male control, 9 male *Kirrel3^Q128A^, n* = 8 female control and 9 female *Kirrel3^Q128A^* mice for **(D)**.

To investigate this, we performed odor preference assays using both attractive and aversive odorants detected by olfactory sensory neurons. First, we tested attraction to peanut butter, a highly palatable odor, across a range of dilutions. Male and female control, *Kirrel3^−/−^*, and *Kirrel3^Q128A^* mice all exhibited robust preference for peanut butter over the solvent control (peanut oil), with preference emerging at approximately a 1:100 dilution (Figures 4A,B). No differences in preference were observed between genotypes at any concentration tested in either male or female mice (Figure 4B; see Supplemental Table S1 for a complete list of statistics), indicating intact detection of appetitive odor cues.

We next assessed responses to the aversive odorant 2-methylbutyric acid (2-MBA), which activates dorsal olfactory bulb circuits associated with innate avoidance (Cho et al., 2011; Horio et al., 2019; Kobayakawa et al., 2007). Mice were presented with swabs containing increasing concentrations of 2-MBA versus water. As expected, increasing concentrations of 2-MBA elicited avoidance behavior, reflected by a negative preference index. However, neither male nor female *Kirrel3^−/−^* nor *Kirrel3^Q128A^* mice differed from controls at any concentration tested (Figures 4C,D; see Supplemental Table S1 for a complete list of statistics).

Together, these results demonstrate that loss of Kirrel3 or disruption of its homophilic adhesion does not impair detection of either attractive or aversive odorants mediated by the main olfactory system. Combined with the observed deficits in urine chemosignal detection, these findings indicate that Kirrel3 is selectively required for normal sensory acuity in the accessory olfactory system.

### 3.5 Differences in social novelty behavior in *Kirrel3^−/−^* and *Kirrel3^Q128A^* mice

**Figure 5:**
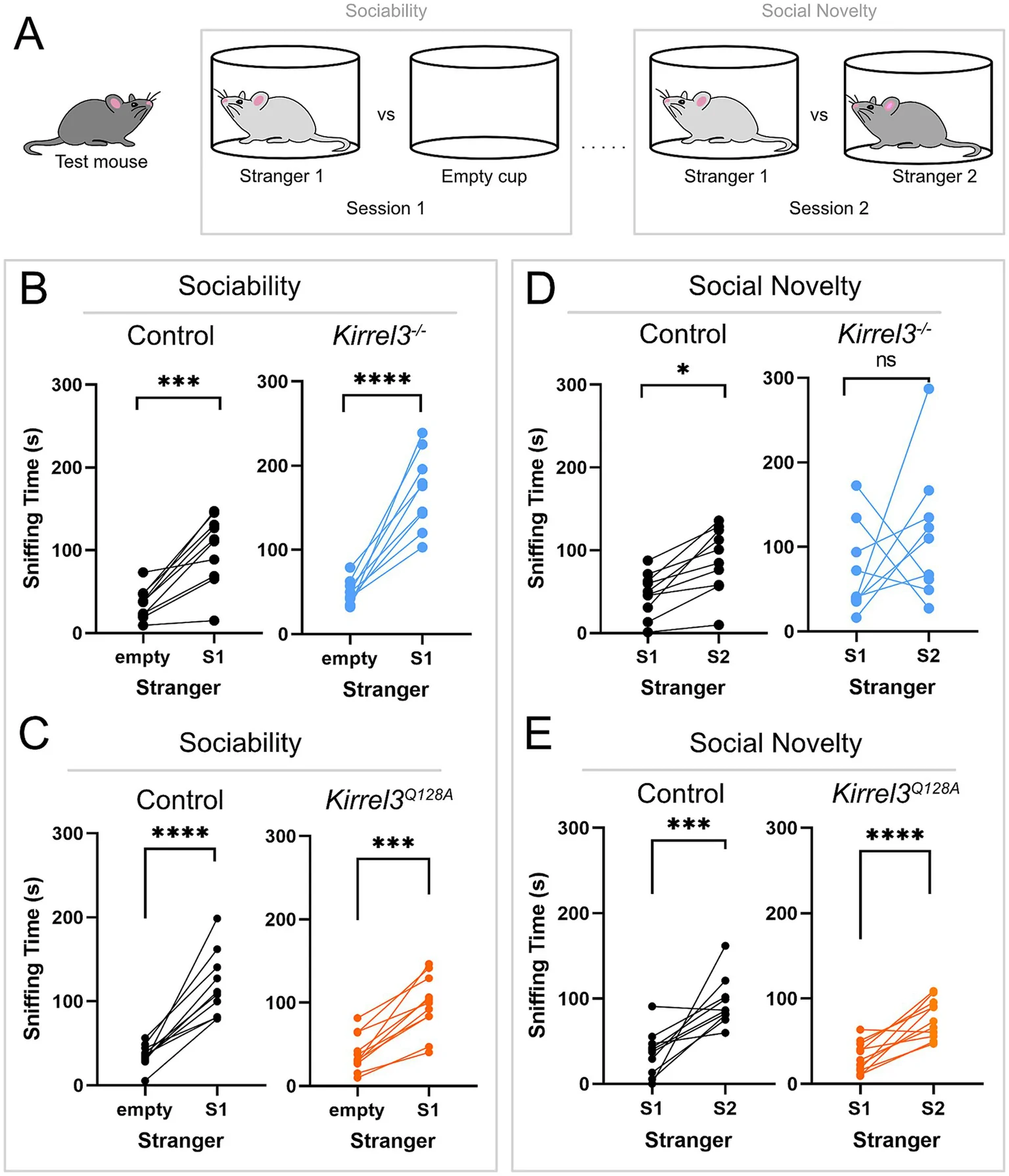
Differences in social novelty behavior, but not sociability, between *Kirrel3^−/−^* and *Kirrel3^Q128A^* mice. **(A)** Schematic of the 3-chamber sociability and social novelty test. **(B)** Sniffing time for session 1 of the 3-chamber sociability test for control and *Kirrel3^−/−^* mice. Both control mice (empty: 35.71 ± 5.78 s, S1: 101.31 ± 13.22 s; ***p = 0.0003; Student’s *t*-test, *t* = 4.54) and *Kirrel3^−/−^* mice (empty: 50.24 ± 4.88 s, S1: 169.60 ± 15.31 s; ****p < 0.0001, Student’s t-test, t = 7.43) spend significantly more time sniffing the “stranger 1” mouse over the empty cup. **(C)** Sniffing time for session 1 of the 3-chamber sociability test for control and *Kirrel3^Q128A^* mice. Both control mice (empty: 35.42 ± 4.34 s, S1: 119.00 ± 12.33 s; ****p < 0.0001; Student’s *t*-test, *t* = 6.39) and *Kirrel3^Q128A^* mice (empty: 39.40 ± 6.69 s, S1: 97.73 ± 10.31 s; ***p = 0.0001; Student’s *t*-test, *t* = 4.74) spend significantly more time sniffing the “stranger 1” mouse over the empty cup. **(D)** Sniffing time for session 2 of the 3-chamber social novelty test for control and *Kirrel3^−/−^* mice. While controls (S1: 46.92 ± 8.28 s, S2: 88.76 ± 12.54 s; *p = 0.0123; Student’s t-test, t = 2.78) spend significantly more time sniffing the “stranger 2” novel mouse over the familiar “stranger 1” mouse, *Kirrel3^−/−^* mice (S1: 71.43 ± 17.53 s, S2: 114.20 ± 26.34 s; ns *p* = 0.1956; Student’s *t*-test, *t* = 1.35) did not show a significant preference for the “stranger 2” mouse. **(E)** Sniffing time for session 2 of the 3-chamber social novelty test for control and *Kirrel3^Q128A^* mice. Both control (S1: 36.13 ± 8.38 s, S2: 95.52 ± 9.03 s; ****p < 0.0001; Student’s *t*-test, *t* = 4.81) and *Kirrel3^Q128A^* mice (S1: 31.39 ± 5.39 s, S2: 76.85 ± 6.82 s; ****p < 0.0001; Student’s *t*-test, *t* = 5.22) spend significantly more time sniffing the “stranger 2” novel mouse over the familiar “stranger 1” mouse. Data is represented as mean ± SEM. *n* = 10 male control and 9 male *Kirrel3^−/−^* mice for **(B,D)**, *n* = 10 male control and 11 male *Kirrel3^Q128A^* mice for **(C,E)**.

Alterations in the human *KIRREL3* gene have been associated with neurodevelopmental disorders, including autism spectrum disorder (ASD), intellectual disability, and Jacobsen syndrome, conditions often characterized by deficits in social behavior and communication (Bhalla et al., 2008; Ciaccio et al., 2021; Dalm et al., 2015; Guerin et al., 2012; Kalsner et al., 2018). To determine whether Kirrel3 loss or disruption of its homophilic adhesion function affects higher order social behaviors in mice, we assessed sociability and social novelty using the three-chamber assay (Figure 5A).

During the sociability phase (session 1), mice were given the choice between an empty cup and a cup containing an unfamiliar conspecific (“stranger 1”). Littermate controls and *Kirrel3^−/−^* mice all spent significantly more time investigating the social stimulus compared to the empty cup (control empty: 35.71 ± 5.78 s, control S1: 101.31 ± 13.22 s, *p* = 0.0003; *Kirrel3^−/−^* empty: 50.24 ± 4.88 s, *Kirrel3^−/−^* S1: 169.60 ± 15.31 s, *p* = <0.0001; Figure 5B). *Kirrel3^Q128A^* and control littermates also spent significantly more time investigating the social stimulus over the empty cup (control empty: 35.42 ± 4.34 s, control S1: 119.00 ± 12.33 s, *p* < 0.0001; *Kirrel3^Q128A^* empty: 39.40 ± 6.69 s, *Kirrel3^Q128A^* S1: 97.73 ± 10.31 s, *p* = 0.0001; Figure 5C), indicating intact sociability behavior. In the social novelty phase (session 2), the empty cup was replaced with a second unfamiliar mouse (“stranger 2”), allowing the test mouse to choose between a familiar and a novel conspecific. Littermate control mice showed a clear preference for the novel mouse, reflecting normal social novelty behavior (S1: 46.92 ± 8.28 s, S2: 88.76 ± 12.54 s, *p* = 0.0123, Figure 5D). In contrast, *Kirrel3^−/−^* mice did not exhibit a preference for the novel over the familiar mouse, indicating a deficit in social novelty (S1: 71.43 ± 17.53 s, S2: 114.20 ± 26.34 s, *p* = 0.1956, Figure 5D). Notably, *Kirrel3^Q128A^* mice, in which Kirrel3 homophilic adhesion is specifically disrupted, retained normal preference for the novel mouse, similar to littermate controls (control S1: 36.13 ± 8.38 s, control S2: 95.52 ± 9.03 s, *p* < 0.0001; *Kirrel3^Q128A^* S1: 31.39 ± 5.39 s, *Kirrel3^Q128A^* S2: 76.85 ± 6.82 s, *p* < 0.0001, Figure 5E).

Together, these results suggest that while Kirrel3 function is not required for baseline sociability, it may contribute to social novelty behavior through a mechanism that is independent of its homophilic adhesion function.

## 4 Discussion

This study demonstrates that Kirrel3-mediated homophilic adhesion is a critical molecular mechanism underlying vomeronasal circuit function and social behaviors driven by chemosensory cues. By combining behavioral assays with a targeted adhesion-disrupting amino acid substitution in Kirrel3 (Kirrel3Q128A), we show that selective impairment of Kirrel3 homophilic interactions is sufficient to recapitulate key phenotypes observed in Kirrel3 null animals, including reduced male–male aggression, diminished maternal aggression, and decreased sensitivity to urinary chemosignals. These findings provide direct evidence that precise Kirrel3 adhesive interactions are critical for the assembly and function of chemosensory circuits underlying aggression.

### 4.1 Kirrel3 homophilic adhesion in aggression circuits

The accessory olfactory bulb (AOB) encodes complex social information through spatially organized glomerular activity patterns. Calcium imaging studies have shown that glomerular maps discriminate not only between male and female urine, but also between juvenile and adult animals (Hammen et al., 2014; Meeks et al., 2010). This encoding reflects the combinatorial detection of diverse chemosignals in urine, including major urinary proteins (MUPs), volatile ligands, sulfated steroids, and other small molecules (Brennan et al., 1999; Isogai et al., 2011). These ligands activate distinct subsets of vomeronasal receptors, primarily V2Rs in basal VSNs, although some compounds also engage V1Rs in apical neurons (Boschat et al., 2002). Given this complexity, disruption of Kirrel3 function could, in principle, affect the formation of either specific subsets of glomeruli (e.g., those tuned to male-specific cues such as MUPs) or the overall organization of the AOB.

Our results confirm that Kirrel3 is essential for male–male aggression and further show that this requirement is specifically mediated through its homophilic adhesion function. *Kirrel3^Q128A^* males phenocopied *Kirrel3^−/−^* mice in the resident–intruder assay, failing to increase aggression in response to male urine cues. A notable finding is that the requirement for Kirrel3 adhesion extends across sex-specific behavioral contexts. Both *Kirrel3^−/−^* and *Kirrel3^Q128A^* postpartum females exhibited reduced maternal aggression, indicating that Kirrel3-dependent circuit assembly is a general feature of aggression-related pathways rather than being male-specific. Furthermore, it suggests that Kirrel3-dependent adhesion is required for global organization of glomerular maps rather than for the formation of circuits dedicated to specific chemosignal classes.

Consistent with this interpretation, both *Kirrel3^−/−^* and *Kirrel3^Q128A^* mice exhibited reduced sensitivity to urinary chemosignals. Mutant animals required higher concentrations of both male and female urine to show preference, indicating a diminished vomeronasal acuity. This deficit was observed in both sexes and across stimulus types, demonstrating that Kirrel3-dependent mechanisms broadly support sensitivity to social chemosignals rather than mediating responses to specific ligands.

These findings provide a mechanistic link between disrupted circuit organization and behavioral output. In intact animals, convergence of similarly tuned VSNs onto discrete glomeruli likely enhances detection sensitivity through signal amplification and noise reduction. Disruption of Kirrel3-mediated adhesion, which results in more dispersed projections, may reduce the coherence of sensory input and elevating detection thresholds (Wang et al., 2021). The fact that mutant mice displayed similar urine preference to controls at higher stimulus concentrations suggests that strong sensory input can partially compensate for circuit disorganization, whereas low-concentration detection critically depends on precise wiring.

### 4.2 Non-homophilic adhesion-dependent functions of Kirrel3

In addition to homophilic adhesion, Kirrel3 undergoes heterophilic interactions with other cell surface receptors that likely contribute to neuronal circuit development and function. For example, Kirrel3 can bind Nephrin in kidney podocytes (Gerke et al., 2005), and related family members (e.g., Kirrel1/Neph1) participate in neural circuit assembly in other systems (Sugie et al., 2010). More recently, Kirrel3 has been shown to interact with IgSF8 in hippocampal neurons (Guo et al., 2025). Single cell RNA sequencing analyses indicate that both *Nephrin (Nphs1)* and *IgSF8* are expressed in VSNs (Devakinandan et al., 2024). Hence, although the phenotypes observed in *Kirrel3^Q128A^* mice are likely to result from the disruption of Kirrel3 homophilic interactions, there remains the possibility that the introduction of the Q128A amino acid substitution disrupts other protein–protein interactions involving Kirrel3. For example, crystal structure data suggests homo- and heterophilic complexes of the *C. elegans* homolog of Kirrel3, SYG-1, are mediated by a shared binding interface with the Nephrin-like SYG-2 (Özkan et al., 2014). Therefore, it remains possible that the Q128A mutation could disrupt heterophilic binding of Kirrel3 with other proteins, which may contribute to the observed behavioral phenotypes.

Our behavioral data also support the possibility that Kirrel3 plays homophilic adhesion-independent roles in neural circuit function. In the three-chamber assay, *Kirrel3^−/−^* mice exhibited impaired social novelty preference, consistent with previous reports (Hisaoka et al., 2018), whereas *Kirrel3^Q128A^* mice displayed normal social novelty behavior when compared to their control littermates. These findings suggest that Kirrel3 contributes to higher order social behaviors through mechanisms that extend beyond homophilic adhesion. Future studies directly comparing social novelty behavior in these two mutant mouse lines would help clarify the extent to which these behavioral differences are attributable to homophilic adhesion-independent functions of Kirrel3.

The hippocampus has been implicated in the facilitation of social novelty preference and recognition (Hitti and Siegelbaum, 2014; Okuyama et al., 2016). Kirrel3 modulates synapse formation and stabilization in the hippocampus. In this context, Kirrel3 regulates the formation of mossy fiber filopodial synapses onto inhibitory interneurons, contributing to feedforward inhibition of CA3 pyramidal neurons (Martin et al., 2015, 2017). Loss of Kirrel3 increases CA3 excitability due to reduced inhibitory input. *In vitro* studies further demonstrate that, although Kirrel3 can promote synaptic differentiation through trans-cellular interactions, not all of its functional effects depend on its ability to homodimerize. Specifically, the introduction of certain amino acid substitutions that do not disrupt Kirrel3 homophilic binding can nevertheless prevent synaptic differentiation, suggesting that Kirrel3 may have homophilic adhesion-independent functions in hippocampal development (Taylor et al., 2020).

In summary, our findings indicate that Kirrel3 homophilic adhesion is an important determinant of vomeronasal circuit function contributing to the sensitive detection of social chemosignals and appropriate aggression responses in both males and females. By distinguishing adhesion-dependent and adhesion-independent roles of Kirrel3, this work provides further insight into how molecular mechanisms of synaptic specificity contribute to the organization of innate behavior and offers a framework for future studies investigating the neural basis of social dysfunction.

## Data Availability

The raw data supporting the conclusions of this article will be made available by the authors, without undue reservation.
