# Supplementary material for: Kirrel3 homophilic adhesion function is required for male-directed aggression and vomeronasal acuity to urine chemosignals
Source: Front Cell Neurosci. 2026 Sep 8;20:1926504. doi: 10.3389/fncel.2026.1926504 (PMC13593424; doi:10.3389/fncel.2026.1926504)
Supplement: Supplementary file 1 [file Data_Sheet_1.PDF]

Supplementary Table 1. Details of statistical tests performed

Figure	Statistical Test	Interaction	DF	MS	F (DFn, DFd)	P value	Corrected P value
1B	2-Way ANOVA with Bonferroni correction	Session	1	1439	F (1, 14) = 50.08	P<0.0001	
		Genotype	1	1111	F (1, 14) = 85.61	P<0.0001	P<0.0001 (left), P>0.9999 (right)
1C	2-Way ANOVA with Bonferroni correction	Session	1	432.4	F (1, 18) = 31.09	P<0.0001	
		Genotype	1	382.5	F (1, 18) = 13.55	P=0.0017	P<0.0001 (left), P=0.5965 (right)
2B	2-Way ANOVA with Bonferroni correction	Session	1	1005	F (1, 15) = 12.12	P=0.0034	
		Genotype	1	1616	F (1, 15) = 4.995	P=0.411	P<0.0001 (left), P>0.9999 (right)
2C	2-Way ANOVA with Bonferroni correction	Session	1	1358	F (1, 19) = 16.38	P=0.0007	
		Genotype	1	4370	F (1, 19) = 10.90	P=0.0038	P<0.0001 (left), P>0.9999 (right)
2D	2-Way ANOVA with Bonferroni correction	Postnatal day	3	36753	F (2.122, 31.83) = 13.19	P<0.0001	
		Genotype	1	14377	F (1, 15) = 3.635	P=0.759	P>0.9999 (P0), P>0.9999 (P1), P=0.6971 (P2), P=0.9262 (P3)
2E	2-Way ANOVA with Bonferroni correction	Postnatal day	3	8730	F (2.244, 33.66) = 2.200	P=0.1212	
		Genotype	1	2836	F (1, 15) = 0.5016	P=0.4897	P>0.9999 (P0), P>0.9999 (P1), P>0.9999 (P2), P>0.9999 (P3)
2F	2-Way ANOVA with Bonferroni correction	Postnatal day	3	0.06376	F (2.374, 42.72) = 0.3242	P=0.7609	
		Genotype	1	2.655	F (1, 18) = 4.055	P=0.0592	P=0.3563 (P0), P>0.9999 (P1), P>0.9999 (P2), P=0.0953 (P3)
2G	2-Way ANOVA with Bonferroni correction	Postnatal day	3	0.05585	F (2.178, 28.31) = 0.2476	P=0.8004	
		Genotype	1	1.5	F (1, 13) = 2.183	P=0.1633	P=0.9599 (P0), P>0.9999 (P1), P>0.9999 (P2), P=0.2394 (P3)
3A	2-Way ANOVA with Bonferroni correction	Urine dilution	4	25831	F (1, 12) = 6.973	P<0.0001	
		Genotype	1	5110	F (12, 48) = 2.081	P=0.0215	P>0.9999 (1:10000), P>0.9999 (1:1000), P=0.0123 (1:100), P>0.9999 (1:10), P=0.3632 (1:1)
3B	2-Way ANOVA with Bonferroni correction	Urine dilution	4	34751	F (2.902, 55.15) = 93.31	P<0.0001	
		Genotype	1	2798	F (1, 19) = 6.897	P=0.0166	P>0.9999 (1:10000), P>0.9999 (1:1000), P=0.0079 (1:100), P=0.4127 (1:10), P>0.9999 (1:1)
3C	2-Way ANOVA	Urine dilution	4	26294	F (2.969, 50.48) = 89.33	P<0.0001	

3C	2-Way ANOVA with Bonferroni correction	Genotype	1	2025	F (1, 17) = 4.411	P=0.0509	P>0.9999 (1:10000),<br>P>0.9999 (1:1000),<br>P=0.0018 (1:100),<br>P>0.9999 (1:10),<br>P>0.9999 (1:1)
3D	2-Way ANOVA with Bonferroni correction	Urine dilution	4	27014	F (3.490, 52.35) = 83.62	P<0.0001	
		Genotype	1	3057	F (1, 15) = 4.054	P=0.0624	P>0.9999 (1:10000),<br>P>0.9999 (1:1000),<br>P=0.0009 (1:100),<br>P>0.9999 (1:10),<br>P>0.9999 (1:1)
3E	2-Way ANOVA with Bonferroni correction	Urine dilution	4	20195	F (3.060, 55.09) = 29.45	P<0.0001	
		Genotype	1	19227	F (1, 18) = 32.30	P<0.0001	P=0.8201 (1:10000),<br>P=0.4397 (1:1000),<br>P=0.0002(1:100),<br>P=0.2050 (1:10),<br>P>0.9999 (1:1)
3F	2-Way ANOVA with Bonferroni correction	Urine dilution	4	34807	F (3.397, 67.94) = 84.44	P<0.0001	
		Genotype	1	2693	F (1, 20) = 5.466	P=0.0299	P>0.9999 (1:10000),<br>P=0.7996 (1:1000),<br>P=0.0145 (1:100),<br>P>0.9999 (1:10),<br>P>0.9999 (1:1)
3G	2-Way ANOVA with Bonferroni correction	Urine dilution	4	23037	F (2.746, 41.20) = 68.62	P<0.0001	
		Genotype	1	2495	F (1, 15) = 2.833	P=0.1130	P=0.5058 (1:10000),<br>P>0.9999 (1:1000),<br>P=0.1002 (1:100),<br>P>0.9999 (1:10),<br>P>0.9999 (1:1)
3H	2-Way ANOVA with Bonferroni correction	Urine dilution	4	24228	F (2.904, 43.57) = 90.96	P<0.0001	
		Genotype	1	1734	F (1, 15) = 2.773	P=0.1166	P>0.9999 (1:10000),<br>P=0.5686 (1:1000),<br>P=0.0130 (1:100),<br>P>0.9999 (1:10),<br>P>0.9999 (1:1)
4A	2-Way ANOVA with Bonferroni correction	Urine dilution	4	40894	F (3.335, 86.71) = 136.3	P<0.0001	
		Genotype	3	1123	F (3, 26) = 2.929	Males:<br>P=0.1121<br>Females:<br>P=0.4998	Males:<br>P>0.9999 (1:10000),<br>P=0.4505 (1:1000),<br>P>0.9999 (1:100),<br>P>0.9999 (1:10),<br>P>0.9999 (1:1)<br>Females:<br>P>0.9999 (1:10000),<br>P>0.9999 (1:1000),<br>P>0.9999 (1:100),<br>P>0.9999 (1:10),<br>P>0.9999 (1:1)
4B	2-Way ANOVA	Urine dilution	4	48303	F (3.780, 124.7) = 99.49	P<0.0001	

4B	2-Way ANOVA with Bonferroni correction	Genotype	3	1299	F (3, 33) = 1.625	Males:<br>P=0.1278<br>Females:<br>P=0.3235	Males:<br>P=0.1679 (1:10000),<br>P>0.9999 (1:1000),<br>P>0.9999 (1:100),<br>P>0.9999 (1:10),<br>P>0.9999 (1:1)<br>Females:<br>P>0.9999 (1:10000),<br>P>0.9999 (1:1000),<br>P=0.8019 (1:100),<br>P>0.9999 (1:10),<br>P>0.9999 (1:1)
4C	2-Way ANOVA with Bonferroni correction	Urine dilution	4	9337	F (2.891, 66.49) = 15.15	P<0.0001	
		Genotype	3	1740	F (3, 23) = 0.8148	Males:<br>P=0.4831<br>Females:<br>P=0.0547	Males:<br>P>0.9999 (1:10000),<br>P>0.9999 (1:1000),<br>P>0.9999 (1:100),<br>P>0.9999 (1:10),<br>P>0.9999 (1:1)<br>Females:<br>P>0.9999 (1:10000),<br>P=0.3563 (1:1000),<br>P>0.9999 (1:100),<br>P>0.9999 (1:10),<br>P>0.9999 (1:1)
4D	2-Way ANOVA with Bonferroni correction	Urine dilution	4	12931	F (2.389, 66.90) = 18.32	P<0.0001	
		Genotype	3	4266	F (3, 28) = 1.319	Males:<br>P=0.9425<br>Females:<br>P=0.7517	Males:<br>P>0.9999 (1:10000),<br>P>0.9999 (1:1000),<br>P>0.9999 (1:100),<br>P>0.9999 (1:10),<br>P>0.9999 (1:1)<br>Females:<br>P>0.9999 (1:10000),<br>P>0.9999 (1:1000),<br>P>0.9999 (1:100),<br>P>0.9999 (1:10),<br>P>0.9999 (1:1)
5B (left)	Unpaired t-test	Genotype	18		t=4.544	P=0.0003	
5B (right)	Unpaired t-test	Genotype	16		t=7.430	P<0.0001	
5C (left)	Unpaired t-test	Genotype	18		t=6.390	P<0.0001	
5C (right)	Unpaired t-test	Genotype	20		t=4.747	P=0.0001	
5D (left)	Unpaired t-test	Genotype	18		t=2.278	P=0.0123	
5D (right)	Unpaired t-test	Genotype	16		t=1.351	P=0.1956	
5E (left)	Unpaired t-test	Genotype	18		t=4.819	P=0.0001	
5E (right)	Unpaired t-test	Genotype	20		t=5.225	P<0.0001	
